# National trends in beverage consumption in children from birth to 5 years: analysis of NHANES across three decades

**DOI:** 10.1186/1475-2891-11-92

**Published:** 2012-10-31

**Authors:** Victor L Fulgoni, Erin E Quann

**Affiliations:** 1 Nutrition Impact, LLC, 9725 D Drive North, Battle Creek, MI, 49104, USA; 2 Dairy Research Institute/National Dairy Council, Rosemont, IL, 60018, USA

**Keywords:** NHANES, Pre-school children, Beverages, Milk, Fruit juice, Fruit drinks, Soft drinks, Nutrient intakes

## Abstract

**Background:**

Given the epidemic of childhood obesity, it is crucial to assess food and beverage intake trends. Beverages can provide a large number of calories and since consumption patterns seem to develop at a young age we examined beverage consumption trends over three decades. The objective of this study was to assess the beverage (milk, fruit juice, fruit drinks, tea, soy beverages, and soft drinks) consumption trends in children <1-5 years of age.

**Methods:**

Data from individuals ages <1-5 years participating in the National Health and Nutrition Examination Survey (NHANES) from 1976–1980, 1988–1994 and 2001–2006 were used to assess beverage consumption and associated calorie and nutrient intakes.

**Results:**

During the NHANES 1976–1980 and 1988–1994 periods, approximately 84–85% of children were consuming milk, whereas only 77% were consuming milk during NHANES 2001–2006. Flavored milk intake was relatively low, but increased to 14% during the last decade (p < 0.001). Fruit juice consumption increased dramatically during NHANES 2001–2006 to more than 50% of the population compared to about 30% in the older surveys (p < 0.001). No significant changes were observed in fruit drink intake across all three decades with 35-37% of this population consuming fruit drinks. At least 30% of children consumed soft drinks. Milk was the largest beverage calorie contributor in all three decades surveyed and was the primary contributor of calcium (52-62%), phosphorus (37-42%), magnesium (27-28%), and potassium (32-37%). Fruit juice and fruit drinks each provided 8-10% of calories with soft drinks providing 5-6% of calories. Fruit juice was an important provider of potassium (16-19%) and magnesium (11%). Fruit drinks provided less than 5% of nutrients examined and soft drinks provided very little of the nutrients evaluated.

**Conclusions:**

Given concerns about childhood obesity and the need to meet nutrition requirements, it is prudent that parents, educators and child caretakers replace some of the nutrient poor beverages young children are currently consuming with more nutrient dense sources like low-fat and fat-free milk.

## Introduction

A child’s first 5 years of life is characterized by rapid growth and development and is the period during which food preferences and behaviors develop that often serve as the foundational basis for future eating habits [1,2]. Beverage consumption patterns developed during the early years can have lasting implications throughout childhood and adolescence and into adult years. The American Academy of Pediatrics (AAP) provides recommendations for the introduction of beverages to children’s diets. They discourage fruit juice intake before the infant is at least 4–6 months of age and fruit drinks, sports drinks and energy drinks are generally discouraged all together [3-5]. The introduction of cow’s milk is recommended after 1 year of age and low-fat and fat-free varieties are encouraged once children turn 2 years old [3]. The 2010 Dietary Guidelines for Americans acknowledge the important link between childhood and adulthood beverage consumption by reinforcing the important habit of drinking milk during childhood because “those who consume milk at an early age are more likely to do so as adults” [6].

In the nationwide sample of infants and toddlers aged 4–24 months who participated in the Feeding Infants and Toddlers Study, beverages provided 36% of the total daily energy [7]. According to 2005–2006 National Health and Nutrition Examination Survey (NHANES) data, total beverage intake among children ages 2–6 years accounts for an average of 367 kcal/day [8]. Although milk intake accounted for the greatest amount of fluids consumed in this young age group (331 ml/day, on average), sodas and fruit drinks provided 206 ml of average daily fluids and by the time children were 7 years of age and older, sodas accounted for the most fluid intake, aside from water, while milk intake continued to decline [8]. The type of beverages consumed can have a significant impact on diet quality and nutrient adequacy.

Lower milk intake makes it more difficult for young children to meet daily milk and milk product group and nutrient intake recommendations, and it could have negative impacts on development since milk and the nutrients it provides are associated with growth and bone health in preschool children [9,10]. Health professionals are concerned that high calorie, high sugar, nutrient poor beverages like soft drinks and fruit-flavored drinks displace nutrient rich beverages such as milk [3]. Sugar-sweetened beverage consumption, primarily referring to carbonated beverages and fruit drinks, begins during the preschool years and generally increases with age [11-13]. The consumption of milk by preschoolers has been positively correlated with diet quality and meeting nutrient recommendations, including vitamin A, folate, vitamin B12, calcium, and magnesium, whereas sugar-sweetened beverage consumption, including soda and fruit drinks, by young children has been linked to reduced milk intake and negatively correlated with achieving intakes of vitamin A, vitamin C, and calcium [14-16].

Research indicates a link between beverage consumption and body weight as well. Childhood overweight and obesity has increased at alarming rates in the United States. Even among children aged 2–5 years of age, obesity defined as body mass index (BMI) greater than the 95th percentile for age, increased from 5 to 10.4% between 1976–1980 and 2007–2008 [17]. There is evidence that a higher intake of sugar-sweetened beverages, such as soda, is associated with increased energy intake [18] and body weight and/or adiposity in children [6,18]. Several studies have observed this phenomenon in preschool children [12,19,20].

In light of the importance of beverage consumption to diet quality and obesity among preschoolers, the purpose of this study was to identify beverage trends in young children over the past three decades. Particular emphasis was placed on evaluating the role of milk and its possible displacement with the rising consumption of empty calorie beverages at this early age.

## Methods

Our analysis utilized beverage intake data available from NHANES 1976–1980, 1988–1994, and 2000–2006 for children less than 1 year of age to 5 years of age. NHANES is an ongoing cross-sectional nationally representative health and nutrition examination survey of a stratified, multistage probability sample of the civilian, non-institutionalized U.S. population [21]. Beverage consumption data was based on 24-hour dietary recalls collected in each NHANES cycle in which proxy respondents reported on all foods and beverages consumed by the children in a 24-hour period. While multiple days of recall were obtained in some NHANES surveys, only one day of recall was used for each subject to allow comparison over time. Participants whose records were incomplete, unreliable, or were being breastfed were excluded. Data from NHANES 1976–1980 included only children 6 months and older, whereas the more recent NHANES included children from birth.

Beverage categories were identified as: milk [total as well as white, flavored (sugar-sweetened), whole, 2%, 1%, and fat-free separately], 100% fruit juice, fruit drinks, soft drinks, tea and soy beverages. White milk included all non-flavored fluid milk consumed as beverages as well as milk consumed with other foods such as cereal and macaroni and cheese. Flavored milk was defined as flavored fluid milk purchased as such (i.e., chocolate, strawberry) or milk prepared by the addition of flavored syrups or powder. Fruit drinks were beverages with less than 100% juice; most contained added sugars. Soft drinks included both “regular” and “diet” sodas. The percentage of the population that consumed each beverage category as well as the amounts consumed by those children was determined by age. The mean intake and percent of total intake of calories, total fat, saturated fat, protein, calcium, phosphorus, magnesium (for NHANES 1988–1994 and 2001–2006 only), and potassium were determined for each beverage category using the respective nutrient content information from each NHANES study period. NHANES 1976–1980 did not measure magnesium intake.

All analyses of percentages and/or means with standard errors were conducted using SAS version 9.2/SUDAAN version 10.0 (SAS Institute, Cary, NC/RTI, Research Triangle Park, NC) to adjust for the complex sampling design of each NHANES survey. Pair-wise comparisons between NHANES 1976–1980 and NHANES 1988–1994, 10–14% NHANES 1988–1994 and NHANES 2001–2006 and NHANES 1976–1980 and NHANES 2001–2006 were performed on mean percents and means using *z* scores to identify significant differences.

## Results

A total sample of 3,998 children <1–5 years of age was utilized from NHANES 1976–1980, 6,871 from NHANES 1988–1994 and 4,430 from NHANES 2001–2006.

### Beverage choice (Percentage use)

**Figure 1:**
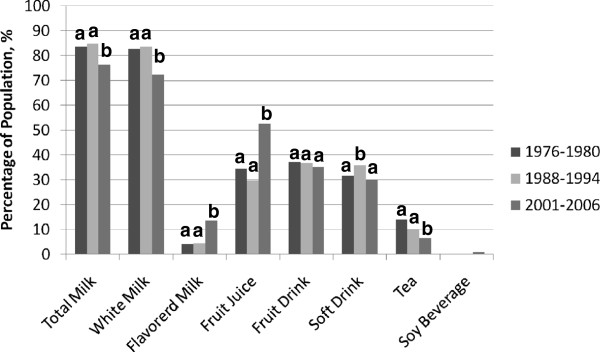
**Percentage of children <1–5 years of age consuming different beverages during NHANES 1976-1980, NHANES 1988–1994, and NHANES 2001–2006.** NHANES – National Health and Nutrition Examination Survey. Columns with different letters within beverage category are significantly different (p < 0.01).

Across all three decades, milk was the beverage consumed by most young children (Figure 1). There was a significant decrease (p < 0.001) in milk consumption in the most recent NHANES surveys compared to the previous surveys. During the NHANES 1976–1980 and 1988–1994 periods, approximately 84–85% of children in this age group were consuming milk, whereas only 77% were consuming milk during NHANES 2001–2006. Flavored milk intake was relatively low during NHANES 1976-1980 and NHANES 1988-1994, but increased to 14% during the last decade (p < 0.001). Fruit juice consumption increased dramatically in this age group during NHANES 2001–2006 to more than 50% of the population compared to about 30% in the older surveys (p < 0.001). No significant changes were observed in fruit drink intake across all three decades. On average, 35-37% of this population consumed fruit drinks. In the case of soft drinks, at least 30% of children consumed this drink on any given day over the past 30 years. During the NHANES 1988–1994 period, there was a significant increase to 36% (p < 0.001) which leveled back to 30% in NHANES 2001–2006. Percent of children consuming tea significantly decreased from 10–14% from previous decades to 7% during NHANES 2001–2006. Consumption of soy beverages were not recorded in the earlier surveys and intake was less than 1% in NHANES 2001–2006. In the remaining analyses, tea and soy beverages were excluded because less than 10% of young children consumed these beverages.

Age differences in beverage trends across decades were assessed in relation to total milk, flavored milk, fruit juice, fruit drink, and soft drinks consumption (Table 1). Among children less than 1 year old, there was an almost 11% increase in milk consumption in NHANES 2001–2006 compared to NHANES 1988–1994. From the age of 1–5 years, the proportion of children that consumed milk was similar for the earlier two decades, whereas during NHANES 2001–2006 there was a tendency for fewer children to consume milk after the age of 3 years. For children 1–3 and 5 years, there was a significant increase (p < 0.05) in the percent of children who were consuming milk during NHANES 1988–1994 compared to NHANES 1976–1980 for nearly all ages. The trend was reversed for NHANES 1988–1994 versus NHANES 2001–2006, where fewer 1, 3 and 5 year olds were consuming milk. Age-related patterns for flavored milk consumption did not differ for the earlier two surveys but showed a significant increase (p < 0.05) ranging from about 1% to 17%, depending on age, during NHANES 2001–2006 compared to NHANES 1988–1994.

**Table 1:** **Percent of children<1**–**5 years that consumed each beverage in NHANES 1976**–**1980**, **NHANES 1988**–**1994**, **and NHANES 2001**–**2006**, **categorized by age**

**Beverage**	**NHANES 1976**-1980* (**A**)	**NHANES 1988**–**1994** (**B**)	**NHANES 2001**–**2006** (**C**)	**Change** (**A**-**B**)	**p**-**value** (**A**-**B**)	**Change** (**B**-**C**)	**p**-**value** (**B**-**C**)	**Change** (**A**-**C**)	**p**-**value** (**A**-**C**)
Total Milk									
<1 year	N/A	3.1 ± 0.7^1^	13.8 ± 1.3	N/A	N/A	10.7	<0.0001	N/A	
1 year	86.0 ± 1.8	90.8 ± 0.9	87.0 ± 1.4	4.8	0.0171	−3.8	0.0224	1.0	0.6610
2 years	82.9 ± 1.6	89.2 ± 1.1	88.3 ± 1.4	6.3	0.0012	−0.9	0.6132	5.4	0.0111
3 years	84.1 ± 1.3	89.4 ± 1.5	83.9 ± 1.8	5.3	0.0076	−5.5	0.0189	−0.2	0.9282
4 years	86.7 ± 1.2	86.5 ± 1.8	83.0 ± 1.4	−0.2	0.9263	−3.5	0.1248	−3.7	0.0448
5 years	86.2 ± 1.6	90.9 ± 1.3	84.0 ± 2.0	4.7	0.0226	−6.9	0.0038	−2.2	0.3904
Flavored Milk									
<1 year	N/A	0.1 ± 0.1	1.2 ± 0.5	N/A	N/A	1.1	0.0310	N/A	
1 year	1.4 ± 0.3	1.8 ± 0.6	6.1 ± 0.9	0.4	0.5510	4.3	0.0001	4.7	<0.0001
2 years	3.8 ± 0.8	2.2 ± 0.6	13.9 ± 1.4	−1.6	0.1096	11.7	<0.0001	10.1	<0.0001
3 years	3.6 ± 0.6	4.6 ± 1.1	15.7 ± 2.0	1.0	0.4248	11.1	<0.0001	12.1	<0.0001
4 years	5.4 ± 1.1	3.2 ± 0.8	19.8 ± 2.2	−2.2	0.1058	16.6	<0.0001	14.4	<0.0001
5 years	8.4 ± 1.1	12.3 ± 1.9	22.2 ± 2.4	3.9	0.0757	9.9	0.0012	13.8	<0.0001
100% Fruit Juice									
<1 year	N/A	31.5 ± 2.4	38.5 ± 2.2	N/A	N/A	7.0	0.0316	N/A	
1 year	36.0 ± 2.3	41.4 ± 2.3	63.3 ± 2.2	5.4	0.0969	21.9	<0.0001	27.3	<0.0001
2 years	36.1 ± 1.9	36.3 ± 2.3	61.3 ± 2.4	0.2	0.9465	25.0	<0.0001	25.2	<0.0001
3 years	32.0 ± 2.2	24.6 ± 1.9	54.7 ± 2.7	−7.4	0.0109	30.1	<0.0001	22.7	<0.0001
4 years	35.1 ± 2.1	25.8 ± 3.2	50.9 ± 2.7	−9.3	0.0151	25.1	<0.0001	15.8	<0.0001
5 years	32.1 ± 2.4	19.9 ± 2.1	41.7 ± 3.6	−12.2	0.0001	21.8	<0.0001	9.6	0.0265
Fruit Drink									
<1 year	N/A	2.9 ± 0.6	7.6 ± 1.0	N/A	N/A	4.7	0.0001	N/A	
1 year	33.4 ± 2.7	29.7 ± 1.7	27.2 ± 1.4	−3.7	0.2462	−2.5	0.2563	−6.2	0.0415
2 years	40.5 ± 2.5	36.5 ± 2.0	35.5 ± 2.2	−4.0	0.2115	−1.0	0.7366	−5.0	0.1348
3 years	41.2 ± 2.2	37.8 ± 2.6	38.7 ± 2.4	−3.4	0.3181	0.9	0.7992	−2.5	0.4426
4 years	40.9 ± 2.1	46.5 ± 2.7	42.3 ± 3.0	5.6	0.1016	4.2	0.2981	1.4	0.7022
5 years	41.8 ± 2.7	44.1 ± 2.8	51.7 ± 2.7	2.3	0.5543	7.6	0.0507	9.9	0.0095
Soft Drinks									
<1 year	N/A	0.6± 0.3	2.4 ± 0.6	N/A	N/A	1.8	0.0073	N/A	
1 year	25.9 ± 2.4	22.7 ± 1.5	18.8 ± 1.7	−3.2	0.2582	−3.9	0.0854	−7.1	0.0158
2 years	34.2 ± 2.2	37.1 ± 2.1	28.9 ± 1.9	2.9	0.3403	−8.2	0.0038	−5.3	0.0683
3 years	37.4 ± 2.2	40.3 ± 3.2	38.3 ± 2.7	2.9	0.4552	−2.0	0.6329	0.9	0.7961
4 years	37.6 ± 1.4	43.0 ± 2.7	41.2 ± 2.8	5.4	0.0758	−1.8	0.6435	3.6	0.2502
5 years	35.1 ± 1.8	46.3 ± 2.2	44.7± 2.5	11.2	0.0001	−1.6	0.6309	9.6	0.0018

*Data only available for children 6 months or older; N/A: not available.

^1^Mean ± standard error; data represent percentage of children consuming any amount of described beverages in the 24-hr recall.

There was a small, but significant decline (p < 0.05) in the percent of 3–5 year olds who consumed fruit juice during NHANES 1988–1994 compared to NHANES 1976–1980. In contrast, there was a significant increase (p < 0.05) in the percent of all children from 1–5 years of age who consumed fruit juice in NHANES 2001–2006. There was a 22-30 percentage unit increase in fruit juice consumption in children 1 year and older. Fruit drink intake on the other hand, was not significantly different between the three decades with the exception of an increase in those less than 1 year of age. It was particularly evident in NHANES 1988–1994 and NHANES 2001–2006 that a greater percent of children consume soft drinks with increasing age. For example, in the most recent survey, approximately 19% consume soft drinks at 1 year of age, 29% at 2 years, 38% at 3 years, 41% at 4 years, and 45% at 5 years. Between NHANES 1976–1980 and NHANES 1988–1994, no clear age differences were present with the exception that there was an 11 percentage unit increase (p < 0.001) in soft drinks consumption among 5 year olds in NHANES 1988–1994. Similarly, no significant age differences were found when NHANES 1988–1994 and NHANES 2001–2006 were compared, except for an increase (p < 0.05) in children less than 1 year and a significant decrease in 2 year olds (p < 0.05).

### Beverage amounts

Overall for the group, there was a small decrease (p < 0.05) in the amount of milk consumed between NHANES 1976–1980 and NHANES 1988–1994 and an increase (p < 0.001) between NHANES 1988–1994 and NHANES 2001–2006 (Table 2). Among 1 year olds there was a small increase (p < 0.05) in the mean amount consumed between NHANES 1976–1980 and NHANES 1988–1994, no significant change in 2 and 3 year olds, and a small to moderate decline in 4 (p < 0.05) and 5 (p < 0.001) year olds. In contrast there was a significant increase in the quantity of milk drank by <1–5 year olds when NHANES 2001–2006 was compared to NHANES 1988–1994. Milk consumption increased from 17.3 to 20.3 fl oz (p < 0.001) in 1 year olds, 13.1 to 16.6 fl oz (p < 0.001) in 2 year olds, 13.3 to 14.9 fl oz (p < 0.05) in 3 year olds, and 13.0 to 15.0 fl oz (p < 0.05) in 5 year olds. No significant changes were observed for 4 year olds. In the most recent survey, 2 and 3 year olds were consuming about 2 cups of milk a day and 4 and 5 year olds were consuming a little less than 2 cups a day.

**Table 2:** **Average amount of each beverage consumed by children<1**–**5 years during NHANES 1976**–**1980**, **NHANES 1988**–**1994**, **and NHANES 2001**-**2006**

**Beverage**	**Mean Intake** (**Fl**. **oz**./**day**)^**1**^			**Change** (**A**-**B**)	**p**-**value** (**A**-**B**)	**Change** (**B**-**C**)	**p**-**value** (**B**-**C**)	**Change** (**A**-**C**)	**p**-**value** (**A**-**C**)
	**NHANES 1976**-1980* (**A**)	**NHANES 1988**–**1994** (**B**)	**NHANES 2001**–**2006** (**C**)						
Total Milk									
<1–5 years	14.7 ± 0.2	14.0 ± 0.2	16.3 ± 0.4	−0.7	0.0133	2.3	<0.0001	1.6	0.0003
<1 year	N/A	22.0 ± 3.4	17.8 ± 1.2	N/A	N/A	−4.2	0.2441	N/A	
1 year	15.3 ± 0.5	17.3 ± 0.6	20.3 ± 0.5	2.0	0.0104	3	0.0001	5	<0.0001
2 years	12.6 ± 0.4	13.1 ± 0.4	16.6 ± 0.5	0.5	0.3768	3.5	<0.0001	4	<0.0001
3 years	13.5 ± 0.4	13.3 ± 0.5	14.9 ± 0.6	−0.2	0.7548	1.6	0.0405	1.4	0.0522
4 years	14.5 ± 0.4	13.2 ± 0.4	13.9 ± 0.4	−1.3	0.0216	0.7	0.2159	−0.6	0.2888
5 years	15.3 ± 0.5	13.0 ± 0.4	15.0 ± 0.7	−2.3	0.0003	2	0.0131	−0.3	0.7273
100% Fruit Juice									
<1–5 years	7.4 ± 0.2	9.5 ± 0.4	10.3 ± 0.3	2.1	<0.0001	0.8	0.1096	2.9	<0.0001
<1 year	N/A	4.8 ± 0.3	5.4 ± 0.3	N/A	N/A	0.6	0.1573	N/A	
1 year	6.6 ± 0.3	9.3 ± 0.8	10.8 ± 0.5	2.7	0.0016	1.5	0.1118	4.2	<0.0001
2 years	8.3 ± 0.4	11.1 ± 0.6	11.0 ± 0.5	2.8	0.0001	−0.1	0.8981	2.7	<0.0001
3 years	7.5 ± 0.4	10.6 ± 1.2	12.2 ± 0.8	3.1	0.0143	1.6	0.2673	4.7	<0.0001
4 years	8.0 ± 0.5	8.8 ± 0.5	9.7 ± 0.4	0.8	0.2579	0.9	0.1599	1.7	0.0079
5 years	8.1 ± 0.5	8.2 ± 0.7	10.0 ± 0.6	0.1	0.9075	1.8	0.0509	1.9	0.0150
Fruit Drink									
<1–5 years	10.4 ± 0.3	10.7 ± 0.3	12.1 ± 0.4	0.3	0.4795	1.4	0.0051	1.7	0.0007
<1 year	N/A	8.1 ± 2.5	8.8 ± 1.2	N/A	N/A	0.7	0.8007	N/A	
1 year	8.8 ± 0.6	9.0 ± 0.6	12.3 ± 0.9	0.2	0.8137	3.3	0.0023	3.5	0.0012
2 years	10.8 ± 0.5	10.0 ± 0.7	12.8 ± 0.8	−0.8	0.3524	2.8	0.0084	2	0.0340
3 years	10.5 ± 0.6	10.7 ± 0.7	10.1 ± 0.4	0.2	0.8283	−0.6	0.4568	−0.4	0.5791
4 years	11.1 ± 0.6	10.6 ± 0.6	12.2 ± 0.9	−0.5	0.5557	1.6	0.1391	1.1	0.3092
5 years	11.1 ± 0.5	12.7 ± 0.8	13.1 ± 0.9	1.6	0.0899	0.4	0.7398	2	0.0521
Soft Drinks									
<1–5 years	7.0 ± 0.3	7.8 ± 0.2	7.9 ± 0.4	0.8	0.0265	0.1	0.8231	0.9	0.0719
<1 year	N/A	6.4 ± 2.8	1.8 ± 0.4	N/A	N/A	−4.6	0.1013	N/A	
1 year	5.4 ± 0.4	5.0 ± 0.3	5.4 ± 1.4	0.4	0.4237	0.4	0.7800	0	1.0000
2 years	6.6 ± 0.3	6.7 ± 0.3	6.2 ± 0.3	0.1	0.8137	−0.5	0.2386	−0.4	0.3458
3 years	6.8 ± 0.3	7.7 ± 0.4	7.6 ± 0.6	0.9	0.0719	0.1	0.8897	0.8	0.2330
4 years	7.9 ± 0.4	8.4 ± 0.4	8.8 ± 0.5	0.5	0.3768	0.4	0.5322	0.9	0.1599
5 years	8.4 ± 0.6	9.7 ± 0.5	9.8 ± 0.6	1.3	0.0960	0.1	0.8981	1.4	0.0990

^1^Mean ± standard error; fluid ounce (fl. oz.). calculated on the assumption that 1 fl oz = 30 g; N/A: not available; 8 fl. oz. is equivalent to one cup.

*Data only available for children 6 months or older.

Fruit juice consumption increased (p < 0.001) in NHANES 1988–1994 compared to NHANES 1976–1980 and then remained stable through NHANES 2001–2006 for the entire sample. Children 1–3 years of age consumed significantly more fruit juice in NHANES 1988–1994 than in NHANES 1976–1980. Among 1, 2, and 3 year olds, mean fruit juice intake increased from 6.6 to 9.3 fl oz (p < 0.05), 8.3 to 11.1 fl oz (p < 0.001), and 7.5 to 10.6 fl oz (p < 0.05), respectively. Changes were not significantly different for 4 and 5 year olds. The change in amounts consumed from NHANES 1988–1994 to 2001–2006 were not significantly different for any of the ages tested. During the NHANES 2001–2006 period, children less than 1 year were consuming 5.4 fl oz, children 1–2 years were consuming about 11 fl oz, children 3 years were consuming 12 fl oz, and children 4–5 years were consuming about 10 fl oz on a daily basis.

The amount of fruit drink ingested did not change significantly for the entire group during the first two decades, but significantly increased (p < 0.05) during the most recent one. None of the age groups demonstrated significant changes in the amount of fruit drink consumed between NHANES 1976–1980 and NHANES 1988–1994. Comparison of NHANES 1988–1994 to NHANES 2001–2006 indicated significant increases from 9.0 to 12.3 fl oz (p < 0.05) and 10.0 to 12.8 fl oz (p < 0.05) for 1 and 2 year olds, respectively. No changes between these surveys were observed for 3–5 year olds. The NHANES 2001–2006 survey found that children less than 1 year consumed 8.8 fl oz of fruit drink, and children 1–5 years consumed about 10–13 fl oz/ day.

Analysis of the group as a whole revealed a small increase (p < 0.05) in the amount of soft drinks consumed between NHANES 1976–1980 and NHANES 1988–1994 as there were trends towards an increase among 3 and 5 years olds. No significant changes in the amounts of soft drinks consumed were noted between NHANES 1988–1994 and NHANES 2001–2006. During the most recent NHANES analysis, children less than 1 year, 1 year, 2 years, 3 years, 4 years, and 5 years consumed 1.8, 5.4, 6.2, 7.6, 8.8 and 9.8 fl oz, respectively (p for trend < 0.0001). Increasing age was associated with increased amounts of soft drinks consumed.

### Beverage calorie and nutrient contributions

Among the four main beverages consumed by children <1–5 years, milk was the largest daily beverage calorie contributor in all three decades surveyed, although there was a significant reduction in the last two surveys compared to NHANES 1976–1980 (Table 3). These calorie contributions include milk used on cereal or in food mixtures too. Milk was the primary contributor for all the macronutrients (Table 3) as well as calcium, phosphorus, magnesium, and potassium (Table 4). About 52%, 55%, and 62% of the daily calcium intake were provided by milk in NHANES 2001–2006, NHANES 1988–1994, and NHANES 1976–1980, respectively. The reduced calcium intake from milk in the more recent surveys is consistent with reduced milk calories. Phosphorus intake from milk ranged from 37-42% across the three decades. Magnesium intake from milk was in the range of 27-28% in the last two surveys. Potassium intake from milk ranged from 31-37%.

**Table 3:** **Energy and macronutrient intake contributed by milk**, **fruit juice**, **fruit drink**, **and soft drinks in the diets of children<1**–**5 years participating in NHANES 1976**–**1980**, **NHANES 1988**–**1994**, **and NHANES 2001**-**2006**

**Nutrient**/**Survey**	**Milk**		**100% Fruit Juice**		**Fruit Drink**		**Soft Drinks**	
	**Mean ± S.E.**	**% Daily Intake**	**Mean ± S.E.**	**% Daily Intake**	**Mean ± S.E.**	**% Daily Intake**	**Mean ± S.E.**	**% Daily Intake**
Calories (kcal/d)								
NHANES 1976-1980	281 ± 5	20.8 ^a^	114 ± 3	8.4 ^a^	128 ± 4	9.0 ^a^	82 ± 3	5.9 ^a^
NHANES 1988-1994	234 ± 4	16.8 ^b^	143 ± 6	10.6 ^b^	129 ± 4	8.7 ^a^	86 ± 3	5.9 ^a^
NHANES 2001-2006	279 ± 6	18.7 ^c^	149 ± 5	9.8 ^c^	150 ± 5	9.4 ^b^	85 ± 4	5.3 ^a^
Total Fat (g/d)								
NHANES 1976-1980	13.5 ± 0.2	25.8 ^a^	0.2 ± 0.0	0.6 ^a^	0.0 ± 0.0	0.0	0.0 ± 0.0	0.0
NHANES 1988-1994	10.9 ± 0.2	21.4 ^b^	0.3 ± 0.0	0.8 ^b^	0.0 ± 0.0	0.1	0.0 ± 0.0	0.0
NHANES 2001-2006	12.0 ± 0.3	22.5 ^b^	0.4 ± 0.0	0.7 ^ab^	0.1 ± 0.0	0.2	0.0 ± 0.0	0.0
Saturated Fat (g/d)								
NHANES 1976-1980	7.5 ± 0.1	26.3 ^a^	0.0 ± 0.0	0	0.0 ± 0.0	0.0	0.0 ± 0.0	0.0
NHANES 1988-1994	6.8 ± 0.2	32.0 ^b^	0.0 ± 0.0	0.3	0.0 ± 0.0	0.1	0.0 ± 0.0	0.0
NHANES 2001-2006	7.2 ± 0.2	32.8 ^b^	0.1 ± 0.0	0.4	0.0 ± 0.0	0.0	0.0 ± 0.0	0.0
Protein (g/d)								
NHANES 1976-1980	16.3 ± 0.3	31.4 ^a^	1.3 ± 0.1	2.9 ^a^	0.0 ± 0.0	0.0	0.0 ± 0.0	0.0
NHANES 1988-1994	13.8 ± 0.2	26.5 ^b^	0.5 ± 0.0	1.2 ^b^	0.1 ± 0.0	0.2	0.0 ± 0.0	0.0
NHANES 2001-2006	15.9 ± 0.4	29.1 ^c^	± 0.0	2.0 ^c^	0.1 ± 0.0	0.3	0.1 ± 0.0	0.2

For each nutrient, different subscripts down a column (a, b, c) indicate statistically significant differences (p<0.05).

**Table 4:** **Calcium**, **phosphorus**, **magnesium**, **and potassium intake contributed by milk**, **fruit juice**, **fruit drink**, **and soft drinks in the diets of children<1**–**5 years participating in NHANES 1976**–**1980**, **NHANES 1988**–**1994**, **and NHANES 2001**–**2006**

**Nutrient**/**Survey**	**Milk**		**100% Fruit Juice**		**Fruit Drink**		**Soft Drinks**	
	**Mean ± S.E.**	**% Daily Intake**	**Mean ± S.E.**	**% Daily Intake**	**Mean ± S.E.**	**% Daily Intake**	**Mean ± S.E.**	**% Daily Intake**
Calcium (mg/d)								
NHANES 1976-1980	553 ± 10	61.7 ^a^	20.6 ± 0.7	3.7 ^a^	29.1 ± 2.2	4.7 ^a^	0.0 ± 0.0	0.0 ^a^
NHANES 1988-1994	504 ± 8	54.5 ^b^	21.8 ± 1.0	3.5 ^a^	31.5 ± 1.5	4.9 ^a^	6.9 ± 0.0	1.3 ^b^
NHANES 2001-2006	565 ± 13	52.3 ^c^	55.2 ± 4.0	5.7 ^b^	34.7 ± 1.9	4.9 ^a^	5.8 ± 0.3	1.0 ^b^
Phosphorus (mg/d)								
NHANES 1976-1980	435 ± 8	41.8 ^a^	32.8 ± 1.2	4.0 ^a^	42.9 ± 3.0	4.7 ^a^	11.0 ± 1.4	1.5 ^a^
NHANES 1988-1994	396 ± 7	37.1 ^b^	22.0 ± 0.9	2.8 ^b^	26.6 ± 1.8	3.0 ^b^	17.1 ± 0.9	2.0 ^b^
NHANES 2001-2006	456 ± 10	39.7 ^c^	31.6 ± 1.1	3.2 ^bc^	11.5 ± 0.7	1.5 ^c^	11.8 ± 0.9	1.8 ^b^
Magnesium (mg/d)								
NHANES 1976-1980	N/A	N/A	N/A	N/A	N/A	N/A	N/A	N/A
NHANES 1988-1994	56.2 ± 0.9	27.8 ^a^	12.9 ± 0.6	7.1 ^a^	5.2 ± 0.2	3.1 ^a^	2.3 ± 0.1	1.5 ^a^
NHANES 2001-2006	53.7 ± 1.3	27.4 ^a^	22.0 ± 0.8	11.1 ^b^	7.6 ± 0.3	4.9 ^b^	1.7 ± 0.1	1.1 ^b^
Potassium (mg/d)								
NHANES 1976-1980	679 ± 13	36.9 ^a^	386 ± 14	18.8 ^a^	26.2 ± 3.4	1.7 ^a^	0.0 ± 0.0	0.0 ^a^
NHANES 1988-1994	645 ± 11	31.4 ^b^	332 ± 14	16.1 ^b^	43.8 ± 2.2	2.7 ^b^	2.6 ± 0.3	0.2 ^a^
NHANES 2001-2006	727 ± 17	33.5 ^c^	436 ± 14	19.2 ^a^	74.9 ± 3.0	4.5 ^c^	4.0 ± 0.3	0.3 ^a^

For each nutrient, different subscripts (a, b) for each survey indicate statistically significant differences (p<0.05).

Percent daily caloric contribution of fruit juice and fruit drink was similar across all three surveys, in the range of 8-11%. (Table 3) Fruit juice was an important provider (16-19%) of potassium in the three surveys and magnesium (11%) in the most recent survey (Table 4). Fruit drinks provided 5% or less of the daily intake of calcium, phosphorus, magnesium, or potassium in all three surveys (Table 4). The caloric contribution of soft drinks in the range of 5-6% did not significantly differ among the three surveys (Table 3). Soft drinks provided very little of the nutrients evaluated (Table 4). The combined calorie contribution from fruit juice, fruit drinks and soft drinks over this thirty year period has increased, but given the parallel rise in total calorie intake the proportion from these beverages has remained relatively consistent at about a quarter of total daily calorie intake (1976–1980: 324 kcals, 23% of total kcal intake; 1988–1994: 358 kcals, 25% of total kcal intake; 2001–2006: 384 kcals, 25% of total kcal intake; data not shown).

**Figure 2:**
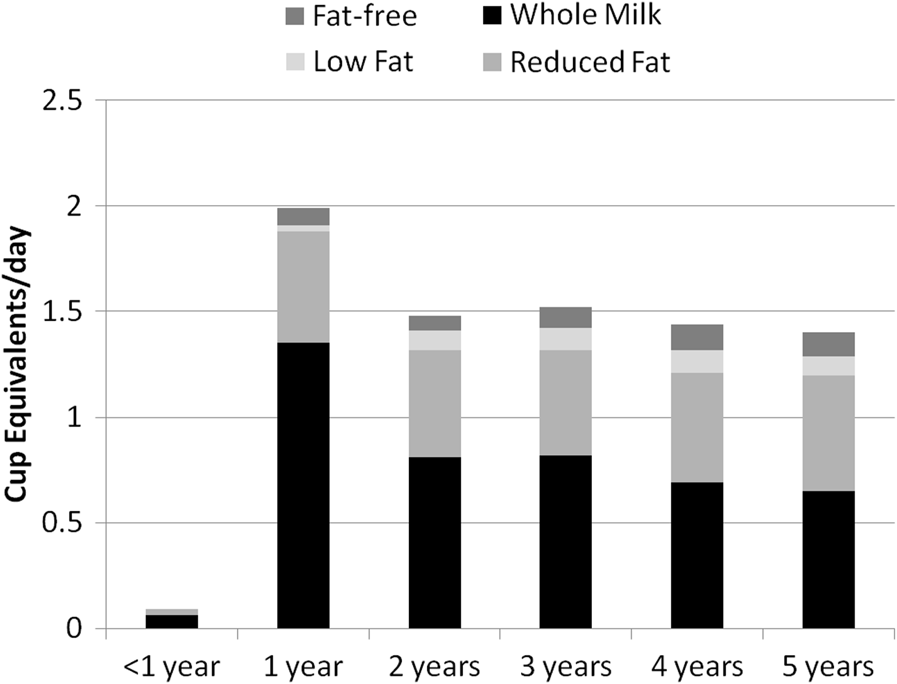
**Servings of milk and type of milk consumed by children <1–5 years of age in NHANES 1988–1994.** NHANES – National Health and Nutrition Examination Survey. Cup equivalents represent 8 oz. units.

**Figure 3:**
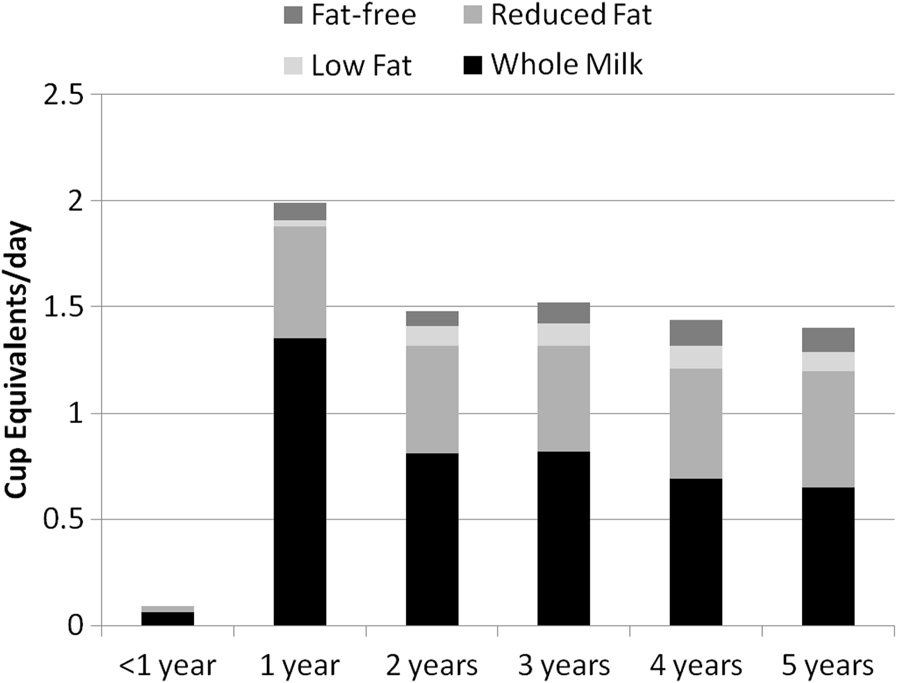
**Servings of milk and type of milk consumed by children <1–5 years of age in NHANES 2001–2006.** NHANES – National Health and Nutrition Examination Survey. Cup equivalents represent 8 oz. units.

Use of different types of milks (Figures 2 and 3) and eating occasion (data not shown) for milk were evaluated in NHANES 1988–94 and NHANES 2001–2006. In both surveys, whole milk was the predominant milk introduced at less than 1 year of age and consumed at 1 year of age. Whole milk intake declined with increasing age in NHANES 2001–2006 compared to NHANES 1988–1994. Two percent or reduced-fat milk was consumed in almost equivalent amounts by age 5 in the most recent survey. One-percent milk and skim milk were consumed in very low amounts for all the years surveyed. Breakfast was the eating occasion where milk was most consumed in both NHANES 1988–1994 and NHANES 2001–2006. Snack time was the next meal occasion in which milk was consumed in both surveys. Slightly less milk was consumed at lunch or dinner than at other meal times. In the context of total dairy intake, relatively small amounts of milk are consumed during meal occasions.

## Discussion

The 2005 Dietary Guidelines for Americans indicated that the nutrients of concern for children were vitamin E, calcium, magnesium, potassium, and dietary fiber [22]. Beverages are a potential source of calcium, magnesium, and potassium, hence these nutrients were evaluated in our study, along with phosphorus, a non-fortified nutrient provided by milk. After our analysis was completed, the 2010 Dietary Guidelines for Americans were released, which noted vitamin D, calcium, potassium, and dietary fiber were nutrients of concern for children above the age of 2 [6]. Milk is the primary source of vitamin D in children’s diets, but it was not possible to evaluate vitamin D trends because vitamin D intake was not assessed in NHANES prior to 2002.

Milk has been and still is the beverage consumed by most preschool children. However, there was a trend towards fewer children consuming milk in the last decade as evidence by this data and previous research [13]. Only 77% of children <1–5 years consumed milk of any type during NHANES 2001–2006 compared to 84-85% in NHANES 1988–1994 and NHANES 1976–1980. To ensure optimal growth and health, the 2010 Dietary Guidelines for Americans recommends children aged 2–3 years drink 2 cups (16 oz) and children aged 4–8 years drink 2 Â½ cups (20 fl oz) of fat-free or low-fat milk or equivalent dairy servings a day [6]. On average 2–3 year olds in NHANES 2001–2006 consumed the recommended amount of milk daily. However, children 4 and 5 years of age were consuming 14–15 fl oz/day; to meet the Dietary Guidelines, recommendation through fluid milk intake alone, 20 fl oz/day would need to be consumed for this age group. Data from Krebs-Smith, et al. indicate about one out of four young children 2–8 years old do not meet milk group recommendations [23]. Although the Dietary Guidelines recommend everyone 2 years of age or older consume low-fat (i.e., 1%) or fat-free milk; the majority of milk currently consumed by 2–5 year olds was whole or 2% milk. Several studies have shown potential health benefits of starting consumption of low-fat/fat-free milk early in life [24,25] and reduced-fat milk would be appropriate for overweight or obese children between 12 months and 2 years of age or those with a family history of obesity, dyslipidemia, or cardiovascular disease [26]. Milk consumption has been reported to increase the likelihood of achieving the recommended intake of calcium by 37% and increase intakes of vitamin A, folate, vitamin B12, and magnesium in 2–5 year olds participating in CSFII 1994–96 [14]. This study found that in addition to calcium and magnesium, milk is a major contributor to the daily intake of phosphorus and potassium in young children. Research has shown young children who drink flavored milk have comparable or higher intakes of many of these nutrients compared to those who exclusively drink plain milk, in part because flavored milk consumers have higher total milk intakes [27].

Over 50% of children 1–5 years consumed fruit juice in NHANES 2001–2006, representing an increase of 23% from the previous decade. Wang et al. [13] found a non-significant increase from 41% to 46% from NHANES 1988–1994 to NHANES 1999–2004 in their study of children 2–5 years of age. Fruit juice was an important source of potassium and magnesium in the diets of these children and has been positively linked to achieving recommended intakes of vitamin C and folate in 2–5 year old children in previous research [14]. Mean daily fruit juice consumption in 2001–2006 was 5.4 fl oz/day for children less than 1 year and 10–12 fl oz/day for children 1–5 years. For the entire group, mean intake was 10.3 fl oz/day in NHANES 2001–2006 compared to 9.5 and 7.4 fl oz/day in NHANES 1988–1994 and NHANES 1976–1980, respectively. Consistent with our findings, Wang et al. [13] observed a significant increase from 9.9 fl oz/day in NHANES 1988–1994 to 11.1 fl oz/day in NHANES 1999–2004 in their preschool sample. This is higher than the fruit juice amount recommended by the AAP, American Heart Association, and Dietary Guidelines (4–6 fl oz/day), and some children were introduced to fruit juice or drinks before the recommended 6 months of age [5,6,28].

In the last three decades, at least 35% of young children consumed fruit drinks, which is similar to the percentage reported by other studies [14,20]. The data indicate a significant increase in the amount of fruit drinks consumed in the last decade, particularly in children 1 and 2 years of age. Children less than 1 year consumed 8.8 fl oz/day and children 1–5 years consumed 10–13 fl oz/day in this analysis of NHANES 2001–2006, which was higher than estimates in CSFII 1994–1996, 1998 (4 fl oz/day) for children 6 months to 6 years [28] and 8 fl oz/day in CSFII 1994–1996 for children 2–5 years. The contribution of calcium, phosphorus, magnesium, and potassium from fruit drinks was negligible. Generally, the amount of fruit drinks consumed was greater than fruit juice for children of all ages analyzed, yet the caloric contribution of fruit drink and fruit juice to these children’s diets was similar. Across all three decades, fruit drinks provided considerably more calories in the diets of preschool children than soft drinks. Although fruit drinks were not a source of the nutrients we measured, many fruit drinks are fortified with vitamin C which was positively associated with achieving recommended intakes of vitamin C in the study by Ballew et al. [14]. Most fruit drinks contain 10% or less fruit juice and a substantial amount of added sugars.

Approximately one-third of young children consumed soft drinks in all three NHANES analyses. These results are consistent with some previous from CSFII 1994–1996 [14], but lower than others including studies of NHANES 1999–2002 (39% 2–5 year olds) [20] and CSFII 1994–1995 (51%) [29]. As children age from 1 year to 5 years, a greater percentage of the population consumed soft drinks. In NHANES 2001–2006, 19% of 1 year olds consumed soft drinks, which increased to 45% by age 5 years. The mean amount of soft drinks marginally increased from 7 fl oz/day in NHANES 1976–1980 to 7.8 fl oz/day in NHANES 1988–1994 and 7.9 fl oz/day in NHANES 2001–2006. These findings are similar to data from CSFII 1994–1996 [14] but appreciably higher than 2.9 fl oz/day indicated by Rampersaud et al. [29] for CSFII 1994–1996, 1998. Carbonated beverages were negatively associated with meeting the recommended levels of vitamin A, vitamin C, and calcium [14].

The cumulative consumption of beverages with added sugar has been inversely linked to overall diet quality and meeting the adequacy of several nutrients [15]. In our analysis of the most recent NHANES, an additional 85 empty calories or 5% of total daily intake was consumed as soft drinks. The decline in milk consumption and the steady intake of nutrient poor beverages such as fruit drinks and soft drinks in our analysis is corroborated by others. Carbonated beverages and added sugar juice drinks were inversely associated with milk intakes in children 1–5 years of age in the Iowa Fluoride Study [15]. Harnack et al. [30] reported in their analysis of CSFII 1994 that high soft drink consumption appears to displace milk and fruit juice in the diets of preschool aged children. For example, those that consumed 9 fl oz or more soft drinks per day were 3.8 times more likely to consume less than 8 oz of milk per day compared to those that did not drink any soft drinks. Although the issue of displacement of milk by other beverages cannot be specifically addressed in cross-sectional data, evidence from longitudinal studies validates this phenomenon. Fiorito and colleagues [31] followed non-Hispanic white girls from age 5–15 years starting in 1996 and observed that early differences in carbonated beverage intake were predictive of later carbonated beverage and milk intake as well as selected nutrients. Girls who consumed carbonated beverages at age 5 years had higher intakes of carbonated beverages, lower milk intake, higher intake of added sugars, lower protein, fiber, vitamin D, calcium, magnesium, phosphorous, and potassium for the 10 years of follow-up. Another study of non-Hispanic white girls who were followed from age 5–9 years observed that girls who consumed adequate amounts of calcium consumed on average twice as much milk, had smaller decreases in milk intake, and consumed 18% less sweetened beverages [32].

The link between beverage consumption, energy intake and body weight have been evaluated in this young population, and is a significant concern because obesity tends to track over time [33]. High intake of sweetened beverages is linked to increased energy intake, weight gain and adiposity in children. Regular consumption of sweetened beverages between meals by children 2.5–4.5 years more than doubled the odds of being overweight at 4.5 years [12]. In a longitudinal study of girls, sweetened beverage consumption (sodas, sport drinks, fruit drinks, and sweetened coffee or tea) at age 5, but not milk or 100% juice, predicted adiposity in childhood and adolescence [19]. Higher intake of sweetened beverages at 5 years was correlated with a higher weight, percent of body fat, and waist circumference from 5–15 years of age [19]. Sweetened beverages in both of these longitudinal studies did not include flavored milk but included sugar-sweetened or artificially sweetened fruit drinks and carbonated beverages. Excessive fruit juice consumption in preschool children has been associated with obesity in some [34] but not all studies [11,35]. Wiley [36] found milk intake was positively associated with BMI among children aged 2–4 years in an analysis of NHANES 1999–2004 data while a cross-sectional analysis of NHANES 1999–2002 data of 2–5 year olds revealed that energy intakes of flavored milk drinkers were higher than those that consumed plain milk or non milk drinkers, but BMI indices did not differ among the three groups [27]. Huh et al. [37] reported in their study that milk intake at age 2 years, whether full or reduced-fat, was not linked to the risk of overweight at age 3 years.

Our analysis was limited by the use of cross-sectional data and associated analytical data across three time periods. But in the case of nationally, representative dietary data, NHANES data is the only source currently available. Given the cross-sectional approach of NHANES causality cannot be determined. Also the data was based on a single 24-hour dietary recall which may not be a fair representation of an individual’s usual intake. The 24-hour dietary recall has been shown to be prone to both under- an over-reporting. Nevertheless, this method has been shown to be valid in estimating the mean intake of a population [38,39].

In summary, the number of young children consuming milk has significantly declined while those consuming fruit juice has increased dramatically in the last decade compared to the previous two decades. The proportion of children that consume fruit drinks and soft drinks has remained high and relatively stable across the three decade time period. Milk is the main source of calcium in the diets of these children, which has been declining alongside increased reduced-fat milk consumption. Milk is also a major source of magnesium and potassium, short-fall nutrients identified by the 2005 and 2010 Dietary Guidelines for Americans. The amount of milk that is consumed in this age group is less than recommended, particularly among 4 and 5 year olds, where fruit juice consumption is significantly higher than recommended. Fruit drinks are a significant source of calories. A greater number of children consume soft drinks as they age from 1–5 years. Since dietary patterns established as young children are carried throughout childhood and adolescence, and a link between non-milk sweetened beverages and obesity has been increasingly demonstrated, it is prudent that parents, educators and child caretakers replace some of the nutrient poor beverages young children are currently consuming with low-fat and fat-free milk.

## Competing interests

VLF as Senior Vice President of Nutrition Impact, LLC, performs consulting and database analyses for various food and beverage companies and related entities. EEQ is an employee of Dairy Research Institute/National Dairy Council.

## Authors’ contributions

VLF designed the study, was primarily responsible for the data analysis, and provided critical input into the manuscript; EEQ helped with data analysis and drafted the manuscript. All authors read and approved the final manuscript.

## Sources of support

Dairy Research Institute administered by the National Dairy Council
